# Epigenetic Dysregulation of the *Homeobox A5* (*HOXA5*) Gene Associates with Subcutaneous Adipocyte Hypertrophy in Human Obesity

**DOI:** 10.3390/cells11040728

**Published:** 2022-02-18

**Authors:** Luca Parrillo, Rosa Spinelli, Mattia Costanzo, Pasqualina Florese, Serena Cabaro, Antonella Desiderio, Immacolata Prevenzano, Gregory Alexander Raciti, Ulf Smith, Claudia Miele, Pietro Formisano, Raffaele Napoli, Francesco Beguinot

**Affiliations:** 1 Department of Translation Medicine, Federico II University of Naples, 80131 Naples, Italy; spinelli.rossella@gmail.com (R.S.); mattcostanzo1595@gmail.com (M.C.); lina.florese@gmail.com (P.F.); serenacabaro@hotmail.com (S.C.); antonella.desid@gmail.com (A.D.); imma987@hotmail.it (I.P.); gregoryraciti@gmail.com (G.A.R.); c.miele@ieos.cnr.it (C.M.); pietro.formisano@unina.it (P.F.); napoli@unina.it (R.N.); 2 URT Genomic of Diabetes, Institute of Experimental Endocrinology and Oncology, National Research Council, 80131 Naples, Italy; 3 Lundberg Laboratory for Diabetes Research, Department of Molecular & Clinical Medicine, Sahlgrenska Academy, University of Gothenburg, 41345 Gothenburg, Sweden; ulf.smith@medic.gu.se

**Keywords:** DNA methylation, adipose tissue, preadipocyte, obesity, transcription factors, T2D familiarity, epigenetic marks, gene expression, human adipogenesis, adipocyte hypertrophy

## Abstract

Along with insulin resistance and increased risk of type 2 diabetes (T2D), lean first-degree relatives of T2D subjects (FDR) feature impaired adipogenesis in subcutaneous adipose tissue (SAT) and subcutaneous adipocyte hypertrophy well before diabetes onset. The molecular mechanisms linking these events have only partially been clarified. In the present report, we show that silencing of the transcription factor *Homeobox A5* (*HOXA5*) in human preadipocytes impaired differentiation in mature adipose cells in vitro. The reduced adipogenesis was accompanied by inappropriate *WNT*-signaling activation. Importantly, in preadipocytes from FDR individuals, *HOXA5* expression was attenuated, with hypermethylation of the *HOXA5* promoter region found responsible for its downregulation, as revealed by luciferase assay. Both *HOXA5* gene expression and DNA methylation were significantly correlated with SAT adipose cell hypertrophy in FDR, whose increased adipocyte size marks impaired adipogenesis. In preadipocytes from FDR, the low *HOXA5* expression negatively correlated with enhanced transcription of the *WNT* signaling downstream genes *NFATC1* and *WNT2B*. In silico evidence indicated that *NFATC1* and *WNT2B* were directly controlled by *HOXA5*. The *HOXA5* promoter region also was hypermethylated in peripheral blood leukocytes from these same FDR individuals, which was further revealed in peripheral blood leukocytes from an independent group of obese subjects. Thus, *HOXA5* controlled adipogenesis in humans by suppressing *WNT* signaling. Altered DNA methylation of the *HOXA5* promoter contributed to restricted adipogenesis in the SAT of lean subjects who were FDR of type 2 diabetics and in obese individuals.

## 1. Introduction

In humans, the expansion of adipose tissue may include increased adipose cell size; i.e., hypertrophy and/or de novo formation of lipid-storing adipocytes from resident adipocyte-precursor cells; i.e., hyperplasia. An elevated ratio of hypertrophic vs. hyperplastic adipose tissue has been reported in different metabolically unhealthy states, including type 2 diabetes (T2D), obesity accompanied by increased risk of T2D, and first-degree relatives of T2D individuals, even when lean and healthy [1]. These observations led to the interpretation that, at least in the subcutaneous adipose tissue (SAT), differentiation of novel adipocytes is of major importance in determining a healthy, as opposed to unhealthy, adipose tissue generation. In particular, limited recruitment of new adipose cells in the SAT of obese individuals has been shown to be accompanied by hypertrophy of the pre-existing adipocytes and lipid spillover in ectopic sites [2], predicting future development of T2D even independent of obesity [3]. In addition, first-degree relatives of T2D patients (FDR) feature hypertrophic SAT leading to an obesity-like metabolic profile with persistent low-grade inflammation, impaired insulin sensitivity, and dyslipidemia [3]. As a consequence, FDR subjects assume an obeselike phenotype even when they are lean [3]. However, the molecular mechanisms favoring healthy accumulation of adipose tissue, rather than excessive adipocyte hypertrophy, have been only in part elucidated.

The class I Homeobox family of transcription factors (*HOX* genes) have a major role in embryonic development [4]. Increasing evidence further indicates that, in humans, the *Homeobox A5* (*HOXA5)* member of the family is differentially expressed in different adipose regions, and plays an active role in regulating both adipocyte biology and body fat distribution [5]. Changes in *HOXA5* expression correlate with adiposity levels and the pattern of body fat distribution [5], and have been proposed to represent a molecular signature of different SAT depots [6]. Our previous work revealed that in mouse 3T3L1 preadipocytes, *Hoxa5* deficiency impairs differentiation into mature adipose cells [7], consistent with the possibility that a similar role is also exerted in humans. However, no study is available addressing the specific role of *HOXA5* in human adipocyte differentiation.

Previously published evidence also demonstrated that *HOXA5* was epigenetically regulated in adipose tissue from obese humans and mice [7,8]. These findings, along with mounting evidence indicating the role of epigenetics in the regulation of adipocyte gene function [9], prompted us to examine the epigenetic regulation of *HOXA5* in human conditions with SAT hypertrophy associated with restricted adipogenesis, such as the FDR of type 2 diabetic individuals and obese subjects. In addition, recent evidence now indicates that in humans, obesity- and T2D-associated epigenetic changes in metabolically active tissues (e.g., adipose tissue) reflect the bloodborne DNA, suggesting that blood may be used to easily access circulating epimarkers of metabolic risk. Accordingly, we explored whether the *HOXA5* epigenetic profile was affected in blood samples of FDR and/or obesity subjects.

Our work revealed a previously undescribed role of *HOXA5* in human adipogenesis. We report that *HOXA5*-disturbed DNA methylation and downregulation restrict adipogenesis in FDR of T2D patients and in obese individuals, which may contribute, in part, to their increased risk of metabolic complications.

## 2. Materials and Methods

### 2.1. Study Participants

#### 2.1.1. First-Degree Relatives of Type 2 Diabetics

Individuals with one ascertained first-degree relative with T2D (FDR; *n* = 12) and 12 subjects with no family history of T2D (controls; CTRL) were selected from the European Network on Functional Genomics of Type 2 Diabetes (EUGENE2) consortium [10]. The recruitment and clinical phenotyping of these subjects have been previously described [9,10]. The study adhered to the Code of Ethics of the World Medical Association (Declaration of Helsinki), and was approved by the Ethical Committee of the Sahlgrenska Academy, University of Gothenburg (ethical approval numbers S655-03 and T492-17). All the participants gave their informed consent.

#### 2.1.2. Obese Subjects

A total of 16 Caucasian individuals (8 obese patients and 8 lean individuals) from an independent cohort recruited at the Federico II University of Naples were included in the replication analysis. The recruitment and clinical phenotyping of these subjects have also been previously described [9,11]. The study adhered to the Code of Ethics of the World Medical Association (Declaration of Helsinki), and was approved by the Ethics Committee of the Federico II University of Naples (ethical approval number 254/17). Informed consent was obtained individually from all of the subjects enrolled in the study.

#### 2.1.3. Human Preadipocyte Isolation, Culture, and Treatment

Adipose tissue biopsies were obtained from FDR and CTRL abdominal SAT. Following careful dissection, adipose tissue specimens were digested with collagenase for 45 min at 37 °C. After digestion, the suspension was centrifuged in two phases: an upper (fat cells) and a lower (SVF cells) phase. The isolated fat cells were filtered through a 250 μm nylon mesh, then washed four times with fresh medium for removal of collagenase. Fat cell size was determined on isolated fat cells as described in [12,13]. Briefly, these data were obtained by direct light microscopy examination, and the mean size of 100 cells in each individual was measured. The isolated SVF cells were washed twice, and the erythrocytes were lysed with 155 mmol/L NH_4_Cl for 5 min before seeding SVF in a 55 cm^2^ petri dish. After 3 days, the inflammatory cells (CD14+ and CD45+ cells) and endothelial cells (CD31+ cells) were removed from the adipocyte precursor cell fraction by immune magnetic separation (Miltenyi Biotech, Bergisch Gladbach, NRW, Germany). The preadipocytes were then cultured with DMEM:F12 medium (LONZA, Portsmouth, NH, USA) supplemented with 10% fetal bovine serum (Thermo Fisher Scientific, Waltham, MA, USA), 2 mmol/L glutamine, 100 U/mL penicillin, and 100 μg/mL streptomycin (LONZA). After 2 weeks, any remaining inflammatory cells were removed by immune magnetic separation as described in [3,14]. Preadipocytes, isolated from control donors, at passages 3–5 were treated with 5-azacytidine (5′AZA; Sigma-Aldrich, St. Louis, MO, USA, #A2385); 5-azacytidine exerts its effect during replication, and therefore, the preadipocytes were cultured to 80% confluence and treated with 10 μM 5′AZA for 2 days.

#### 2.1.4. Adipogenic Differentiation of Human Preadipocytes

Preadipocytes were grown and allowed to differentiate into mature adipocytes as described in [14,15]. Preadipocytes were induced to differentiate after 3 days of confluence (at differentiation day 0) with a cocktail consisting of 850 nmol/L insulin, 10 μmol/L dexamethasone (Sigma-Aldrich), 0.5 mmol/L isobutylmethylxanthine (MP Biomedicals, Irvine, CA, USA), 10 μmol/L rosiglitazone (Cayman Chemical, Ann Arbor, Michigan, USA) in DMEM/F12 supplemented with 3% FBS, 2 mmol/L glutamine, and antibiotics. After 3 days, the medium was replaced with adipocyte medium consisting of 850 nmol/L insulin, 1 μmol/L dexamethasone, 1 μmol/L rosiglitazone in DMEM/F12 supplemented with 10% FBS, 2 mmol/L glutamine, and antibiotics. The adipocyte medium was changed every 3 days throughout the differentiation period until day 15. To determine lipid accumulation, differentiated preadipocytes (at differentiation day 15) were fixed with 4% formaldehyde for 5 min at room temperature and stained with Oil Red O (Sigma-Aldrich, #O1391) as previously reported [15].

#### 2.1.5. Silencing of *HOXA5* in Human Preadipocytes

Human SAT preadipocytes were grown and were allowed to differentiate into mature adipocytes as described above. For *HOXA5* silencing, preadipocytes were transfected with 25 nmol/L of *HOXA5* human siRNA (OriGene, Rockville, MD, USA, #SR302182) at differentiation day −1, and subsequently induced to differentiate into mature adipocytes. Transfection was repeated as on day −1 every 48 h until day 15. Then, lipid accumulation was assessed by Oil Red O staining as previously described [15].

#### 2.1.6. Total RNA Isolation and Quantitative Real-Time PCR

Total RNA was extracted using the AllPrep DNA/RNA Mini Kit (Qiagen, Germantown, MD, USA, #80224). The cDNA synthesis was performed as previously described [16]. The qPCR reactions were run with SYBR Green PCR Master Mix (Bio-Rad, Hercules, California, USA, #1725124) as previously indicated [17]. Primers are shown in the Supplementary Materials (Table S1). Human *RPL13* was used as reference genes. The qPCR conditions were as follows: 95 °C for 30 s, 40 × (95 °C for 5 s and 60 °C for 30 s). All reactions were run in triplicate on a QuantStudio 7 Flex Real-Time PCR System (Thermo Fisher Scientific).

#### 2.1.7. RT^2^ Profiler PCR Array

The expression of 84 genes related to *WNT*-mediated signal transduction were analyzed using the Human *WNT* Signaling Pathway RT^2^ Profiler PCR Array (PAHS-043ZD; SABiosciences, Qiagen). According to the manufacturer’s protocol, real-time PCR was performed using RT^2^ Profiler PCR Arrays in combination with RT^2^ SYBR Green/ROX PCR Master Mix (Qiagen). The expression levels were quantified relative to the values obtained for housekeeping genes (*ACTB*, *B2M*, *GAPDH*, *HPRT1*, and *RPLP0*). Data analyses were performed using web-based analysis software (http://pcrdataanalysis.sabiosciences.com/pcr/arrayanalysis.php, accessed on 1 July 2020).

#### 2.1.8. Bisulfite Sequencing

Bisulfite treatment of genomic DNA (gDNA) extracted with the AllPrep DNA/RNA Mini Kit (Qiagen) was performed using the EZ DNA Methylation Kit (Zymo Research, Irvine, CA, USA, #D5002). Converted gDNA was amplified by PCR using specific primers for the *HOXA5* promoter region. Bisulfite sequencing (BS) was performed as previously reported [9,18] on an ABI 3500 Automatic Sequencer by using Big-Dye Terminator v3.1 (Applied Biosystem, Foster City, CA, USA, #4336917). Primers for BS were designed by MethPrimer (http://www.urogene.org/methprimer, accessed on 1 July 2020), and are shown in Table S1.

#### 2.1.9. Luciferase Assay

A 505 bp DNA fragment (−565 bp to −60 bp from TSS) corresponding to the *HOXA5* minimal promoter and containing 36 CpG sites was amplified by PCR and cloned into a CpG-free basic firefly luciferase reporter vector (pCpG-basic; InvivoGen, Toulouse France, #pCpGf-basIc). Primers are shown in Table S1. Luciferase assays were carried out as reported in [9]. In vitro methylation was performed using the *M.SssI* CpG methyltransferase (New England Biolabs, Ipswich, Massachusetts, USA, #M0226L) and S-adenosylmethionine (SAM; New England Biolabs, #B9003S), following the manufacturer’s instructions. Unmethylated constructs were treated as the methylated construct, including application of SAM, but in the absence of *M.SssI* (mock-treated). In vitro methylation was confirmed by resistance to *HhaI* or *HpaII* (New England Biolabs) digestion. Constructs were transfected into ChubS7 cells (immortalized human preadipose cell line) by lipofectamine (Thermo Fisher Scientific, #L3000-015), following the manufacturer’s instructions. Transfection of ChubS7 with the mock-treated empty vector was used to control for background firefly luciferase activity. Firefly luciferase activity of each transfection was normalized against renilla luciferase activity (Promega, Madison, WI, USA, #E2231).

#### 2.1.10. Peripheral Blood Leukocyte Isolation

Peripheral blood leukocytes (PBLs) were isolated as previously described [11]. Briefly, 1 mL of whole blood sample were incubated on ice for 15 min with 5 mL of erythrocyte lysis buffer (KHCO3 10 mM, NH4Cl 155 mM, EDTA 0.1 mM), and PBLs were recovered by centrifugation at 400× *g* for 10 min. PBLs obtained from each patient were lysed in RLT buffer (Qiagen), and gDNA was then isolated using the AllPrep DNA/RNA Mini Kit (Qiagen) following the manufacturer’s instructions.

#### 2.1.11. Western Blot

Protein extracts were prepared in ice-cold lysis buffer as previously described in [15]. Protein concentration was assessed using the protein assay based on Bradford’s method (Bio-Rad). Total cell extracts in equal amounts were separated by SDS-PAGE and blotted on nitrocellulose membrane (Millipore, Burlington, Massachusetts, USA, #10600001). Upon incubation with primary antibodies against HOXA5 (Abcam, Cambridge Biomedical Campus Cambridge, UK, #ab82645), WNT2B (Abcam, #ab178418), NFATC1 (Cell Signalling, Trask Lane Danvers, MA, USA, #8032), and vinculin (Santa Cruz, Dallas, Texas, USA, #sc-73614,), as well as secondary antibodies (Bio-Rad, #170-6516; #170-6515), immunoreactive bands were detected with an enhanced chemiluminescence kit (Bio-Rad #170-5060) and quantified with the ImageJ 1.53e software. Protein abundance was calculated after vinculin normalization.

#### 2.1.12. Statistical Analysis

The experimental data are shown as mean ± SD. The Mann–Whitney U-test was used to compare anthropometric and biochemical variables, gene expression, and DNA methylation data between the groups. A two-tailed Student’s *t*-test or a one-way analysis of variance (ANOVA) followed by Tukey’s multicomparison test were used, as appropriate, for the in vitro data. The group size (n) for each experimental determination is reported in the figure legends. The value of n refers to either the number of independent biological samples/individuals per group (in vivo analyses) or the number of independently repeated experiments, each performed at least in triplicate (in vitro studies). The correlation between the quantitative variables was tested using Spearman’s rank correlation test. Differences were considered statistically significant at *p* < 0.05. The statistical analyses were performed using the GraphPad software (version 6.01).

## 3. Results

### 3.1. Altered Epigenetic Regulation of HOXA5 Was Associated with Impaired Adipogenesis in FDR Individuals

**Figure 1:**
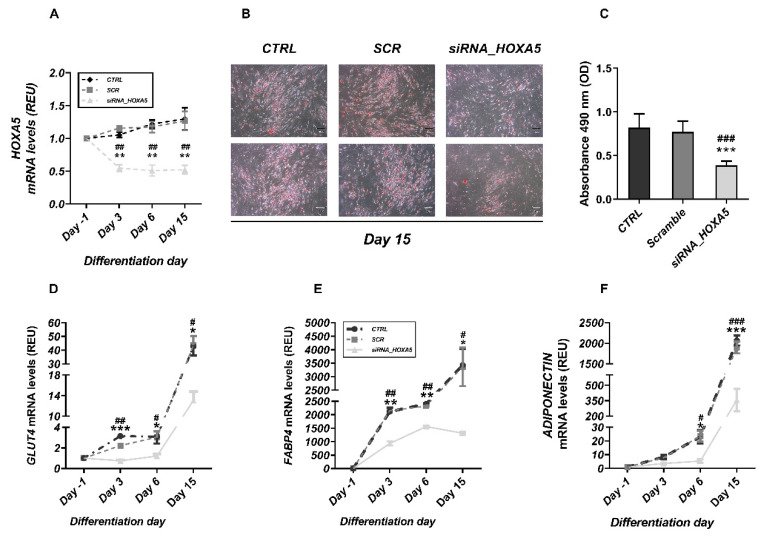
Effect of HOXA5 silencing on the differentiation of human preadipocytes. (**A**) *HOXA5* mRNA levels were measured by qPCR at different time points during differentiation in cells treated with *HOXA5*-specific siRNA (*siRNA_HOXA5*), scrambled siRNA (*SCR*), or control (CTRL). All data are presented as means ± SD of 3 independent experiments. Statistical significance was tested by one-way ANOVA followed by Tukey’s multicomparison test at each indicated time. ** *p* < 0.01 versus *CTRL*; ## *p* < 0.01 versus *SCR*. (**B**) *CTRL* cells and cells treated with *SCR* or *siRNA_HOXA5*, at differentiation day 15, were stained with *Oil Red O* and then subjected to phase contrast microscopy. Representative fields are presented. (**C**) The lipid quantitation was determined by measuring absorbance at 490 nm as described in the Materials and Methods section. Expression levels of *GLUT4* (**D**), *FABP4* (**E**), and *ADIPONECTIN* (**F**) were assessed by qPCR at the indicated times during differentiation. All data are presented as means ± SD of 3 independent experiments. Statistical significance was tested by one-way ANOVA followed by Tukey’s multicomparison test at each indicated time point: * *p* < 0.05; ** *p* < 0.01; *** *p* < 0.001 for comparison versus *CTRL*; # *p* < 0.05, ## *p* < 0.01, ### *p* < 0.001 versus *SCR*.

We first examined whether *HOXA5* has a functional role in human adipogenesis. To explore this possibility, *HOXA5* was silenced in SAT-derived human preadipocytes one day before adipogenic induction (Figure 1A and Figure S1). Based on Oil Red O staining, *HOXA5* silencing significantly reduced lipid accumulation, revealing attenuation of the differentiation process (Figure 1B,C). To further investigate whether *HOXA5* is required for the expression of adipogenesis-related genes, total mRNA was collected at different time points after induction of differentiation, and *GLUT4*, *FABP4,* and *ADIPONECTIN* mRNA levels were analyzed by qRT-PCR. As shown in Figure 1D–F, the expression of these genes was significantly reduced in the *HOXA5*-silenced cells compared to the cells that had been transfected with scrambled-siRNA, indicating that *HOXA5* was required for human adipocyte differentiation. Moreover, during the differentiation process, the *HOXA5*-silenced cells, when compared to the scramble-treated cells, achieved a significantly lower reduction in mRNA expression of *GATA2* and *GATA3*, which are responsible for maintaining the preadipocytes in an undifferentiated state [19]. On the contrary, no significant differences were found in the expression of apoptosis marker FAS between the two cell groups (Figure S2). Taken together, these data suggested that a loss of *HOXA5* function can lead to an impaired expression of other genes enabling a repression of adipocyte differentiation.

**Figure 2:**
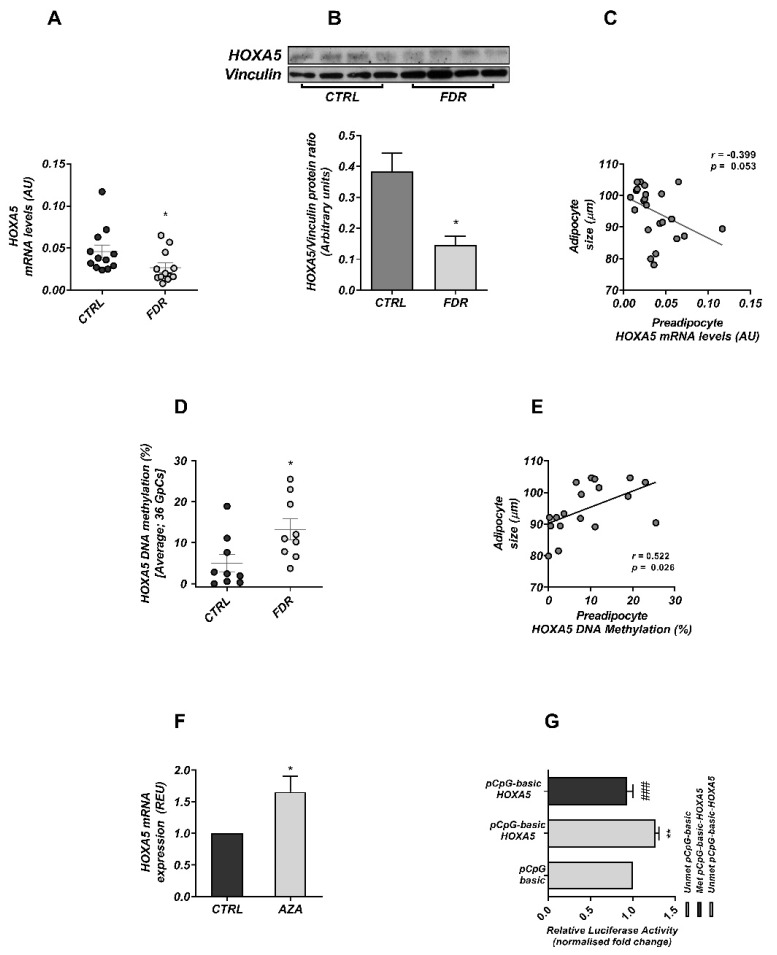
*HOXA5* mRNA expression and DNA methylation in preadipocytes and correlation with the cell size of mature subcutaneous adipocytes from FDR and CTRL subjects. (**A**) The *HOXA5* mRNA expression was measured by qPCR in preadipocytes from FDR (n = 12) and control (CTRL; n = 12) subjects as described in the Materials and Methods section. Data points represent absolute units (AU) from each individual subject. (**B**) *HOXA5* protein levels were assessed by WB in preadipocytes from FDR (n = 4) and CTRL (n = 4) subjects available from the study group. Mean values ± SD are also shown. * *p* < 0.05 in a two-tailed Mann–Whitney U-test. (**C**) The correlation between the preadipocyte *HOXA5* mRNA levels and adipose cell size was assessed in FDR (n = 12) and CTRL (n = 12) subjects. The *r* correlation coefficient and *p*-value are indicated in the graph. (**D**) Changes in average DNA methylation levels at 36 CpG within the *HOXA5* promoter were detected by BS in preadipocytes from FDR (n = 9) and CTRL (n = 9) subjects available from the study group. Mean values ± SD are also shown. * *p* < 0.05 in two-tailed Mann–Whitney U-test. (**E**) Correlation between preadipocyte HOXA5 DNA methylation and mature adipose cell size in FDR (n = 9) and CTRL (n = 9) subjects. The *r* correlation coefficient and *p*-value are indicated in the graph. Effect of DNA methylation on *HOXA5* transcriptional activity. (**F**) *HOXA5* mRNA expression levels in preadipocytes maintained in the absence (CTRL) or the presence of 5′AZA (AZA), as described in the Materials and Methods section, were analyzed by qPCR and normalized to *RPL13*. Results are expressed as the mean ± SD of n = 3 independent experiments. * *p* < 0.05 in a two-tailed Student’s *t*-test. (**G**) A CpG-free basic plasmid with a luciferase reporter gene (pCpG-basic), where the *HOXA5* minimal promoter sequence (containing 36 CpG sites; −60 bp to −565 bp from TSS,) was cloned upstream of the luciferase gene (pCpG-basic-*HOXA5*), was engineered. This construct was then methylated in vitro (Met) or mock-treated (Unmet) and transfected in ChubS7 cells, as reported in the Materials and Methods section. The relative luciferase activity was normalized against the activity of a cotransfected internal vector. The results are the mean ± SD of three independent experiments. The statistical significance was tested by one-way ANOVA followed by Tukey’s multicomparison test: ** *p* < 0.01 versus pCpG-basic; ### *p* < 0.001 versus unmethylated pCpG-basic-*HOXA5*.

To gain further insights into the role of *HOXA5* in human adipose tissue development, we investigated *HOXA5* gene function in a well-established condition of restricted adipogenesis. First-degree relatives of Type 2 diabetics (FDR) feature a dysfunctional SAT accompanied by adipocyte hypertrophy and impaired differentiation of the resident preadipocytes (Table S2); a detailed description of this cohort is provided in [3,9]. We therefore investigated whether the expression of *HOXA5* is also impaired in the preadipocytes isolated from their SAT. Interestingly, *HOXA5* mRNA and protein levels were significantly decreased in the preadipocytes from FDR compared to those from individuals who were not FDR (Figure 2A,B). Reduced *HOXA5* expression in the FDR individuals negatively correlated with their adipocyte hypertrophy (Figure 2C).

Transcriptional repression of *HOXA5* in the FDR may reflect enhanced DNA methylation in the promoter region. Indeed, our recent findings in mice have shown *Hoxa5* hypermethylation and repression in response to high-fat-diet treatment [7]. To test this hypothesis, we adopted bisulfite sequencing and analyzed DNA methylation levels at *the HOXA5* promoter, comparatively, in preadipocytes from individuals who were FDR of type 2 diabetics and in those who had no diabetes familiarity. To this end, *HOXA5* promoter methylation was examined in a region containing 36 CpG sites, located within 600 bp upstream of the transcription start site, as this region has already been reported to regulate *HOXA5* expression in human cancer [20]. Methylation levels at the *HOXA5* promoter were significantly increased in the preadipocytes of the FDR compared to the CTRL subjects (Figure 2D and Figure S3) Importantly, the *HOXA5* methylation pattern was inversely correlated to its downregulation (Figure S4), suggesting that methylation at this locus also repressed *HOXA5* expression in these subjects. These methylation events positively correlated with adipose cell size in the subjects (Figure 2E), suggesting that alterations in DNA methylation at the *HOXA5* promoter contributed to the restricted adipogenesis reported in FDR individuals. [21].

### 3.2. DNA Methylation Modulates HOXA5 Promoter Activity

To further investigate the transcriptional effect of *HOXA5* promoter hypermethylation, we exposed preadipocytes to the demethylating agent 5-AZA [21]. As shown in Figure 2F, we observed that treatment with 5-AZA effectively rescued *HOXA5* mRNA expression in these cells.

We then used a CpG-free basic plasmid with a luciferase reporter gene (pCpGL) where the *HOXA5* minimal promoter sequence (containing 36 CpG sites; −60 to −565 bp from TSS) was cloned upstream of the luciferase gene. This construct was methylated in vitro or mock-treated and transfected in ChubS7 cells. In the luciferase assay, the unmethylated construct with the *HOXA5* promoter region (unmet pCpG-basic-*HOXA5*) revealed a higher luciferase activity compared to the mock-treated empty vector (unmet pCpG-basic). In contrast, the methylated *HOXA5* promoter construct (met pCpG-basic-*HOXA5*) exhibited a significantly decreased luciferase activity, by 27%, compared to the unmethylated pCpG-*HOXA5*-promoter construct (Figure 2G). Taken together, these results provide evidence that DNA methylation at this locus exerted a repressive effect on *HOXA5* expression.

### 3.3. HOXA5 Promoted Adipocyte Differentiation by Inhibiting the WNT Signaling Pathway

**Figure 3:**
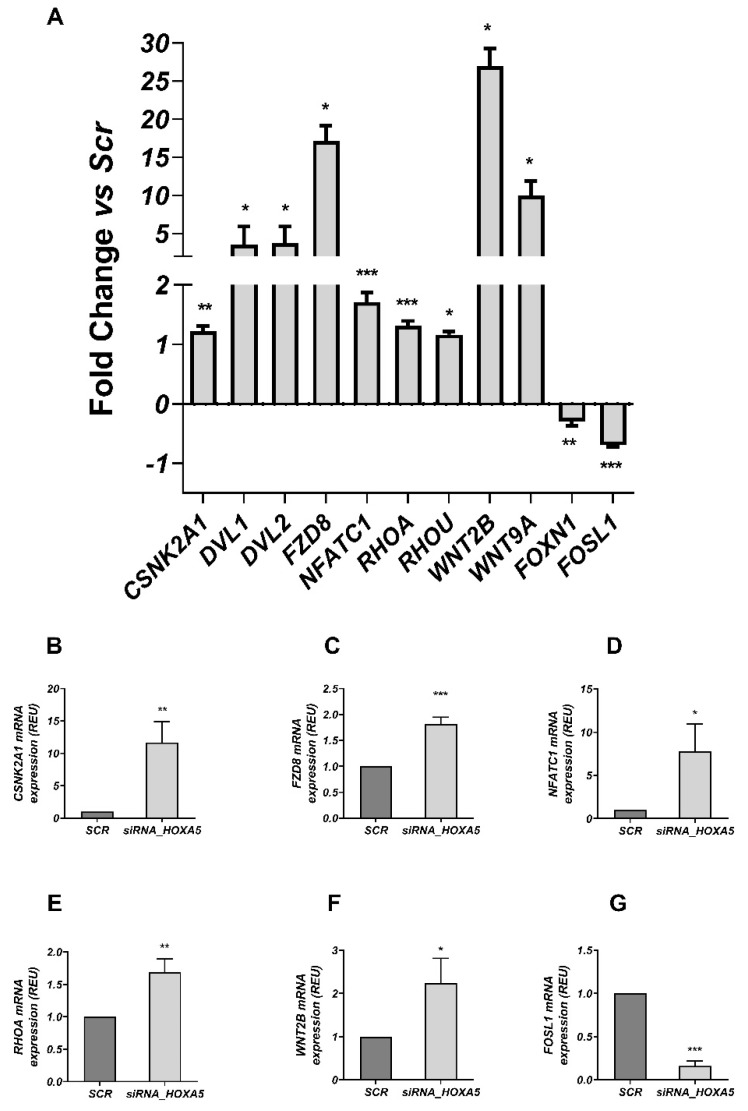
Differential expression of genes embedded in the WNT-signaling pathway in human preadipocytes upon *HOXA5* silencing (PCR array PAHS-043ZD). (**A**) Eighty-four WNT-related genes were analyzed using the RT2 Profiler™ PCR Array (n = 3 per group/condition). The expression of 11 genes changed significantly in the *HOXA5*-silenced preadipocytes compared to cells transfected with scrambled siRNA (SCR). Data analyses were performed using web-based analysis software as described in the Materials and Methods. Data are presented as a fold-change relative to scramble-treated cells. Levels of significance were section: * *p* < 0.05; ** *p* < 0.01; *** *p* < 0.001. The 11 differentially expressed genes, identified by the RT2 Profiler™ PCR array, were individually analyzed by qPCR in the same samples also tested in the PCR array (n = 3 per group/condition). (**B**–**G**) Six of 11 differentially expressed genes were confirmed by qPCR assay. All data are expressed as means ± SD. The statistical significance was tested by two-tailed unpaired *t*-test. * *p* < 0.05; ** *p* < 0.01; *** *p* < 0.001 versus SRC.

In order to identify mechanisms responsible for *HOXA5* function in adipogenesis, we focused on the *WNT*-signaling genes that have been identified as potential targets of *HOXA5* [22,23] and that restrain adipocyte differentiation [24]. To explore this hypothesis, we examined the expression of 84 *WNT* pathway genes in the *HOXA5*-silenced preadipocytes. Nine of these genes were upregulated (*WNT2B*, *FZD8*, *WNT9A*, *DVL1*, *DVL2*, *NFATC1*, *RHOA*, *RHOU* and *CSNK2A1*) in the *HOXA5*-silenced compared to the scrambled cells, with two further genes exhibiting downregulation (*FOSL1* and *FOXN1;* Figure 3A). These differentially expressed genes were further validated by RT-qPCR analysis. Consistent with the array data, the *FZD8*, *NFATC1*, *CSNK2A1*, *WNT2B* and *ROHA* mRNA levels were found to be significantly increased, while attenuation of *FOSL1* mRNA levels was observed upon *HOXA5* silencing in the preadipocytes (Figure 3B–G). No significant variation was found in the expression of *DVL1*, *DVL2*, *FOXN1*, *RHOU,* and *WNT9A* between the *HOXA5*-silenced and scrambled cells (Figure S5). Furthermore, as a proof of concept, the protein amount of the two *WNT*-signaling factors, namely *NFATC1* and *WNT2B*—validated by qPCR as described above—was also increased in the *HOXA5*-silenced preadipocytes compared to the scrambled cells (Figure S6). These findings led us to hypothesize that reduced *HOXA5* function in preadipocytes would result in impaired adipogenesis by deranging the *WNT*-signaling pathway.

**Figure 4:**
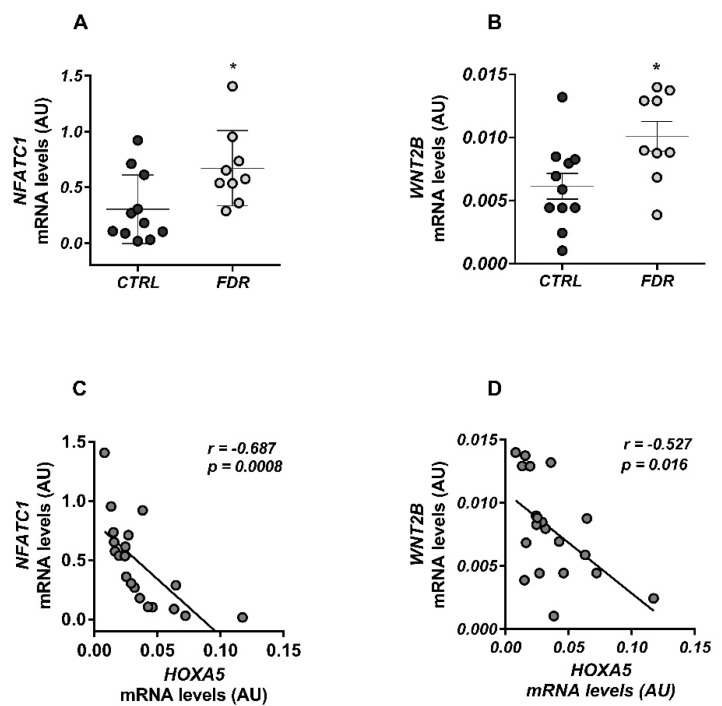
*NFATC1* and *WNT2B* expression and their correlation with *HOXA5* mRNA levels in preadipocytes from FDR and CTRL subjects. (**A**,**B**) The *NFATC1* and *WNT2B* mRNA expression was measured by qPCR in preadipocytes from FDR (n = 9) and control (CTRL; n = 11) subjects available from the study group. Data points represent arbitrary units (AU) from each individual subject. Mean values ± SD are also shown. * *p* < 0.05 in a two-tailed Mann–Whitney U-test. (**C**,**D**) Correlation between the preadipocyte *NFATC1* and *WNT2B* mRNA and *HOXA5* gene expression in FDR (n = 9) and CTRL (n = 11) subjects available from the study group. The *r* correlation coefficient and *p*-value are indicated in the graph.

We have reasoned that these mechanisms may also occur in FDR preadipocytes, in which the low *HOXA5* expression marks impaired adipogenesis. Accordingly, we measured mRNA levels of the *WNT* genes that emerged from the array search in the FDR and control groups. These further studies revealed that the mRNA levels of *NFATC1* and *WNT2B* were upregulated in FDR preadipocytes compared to cells obtained from control individuals (Figure 4A,B). *HOXA5* expression negatively correlated with that of the *NFATC1* and *WNT2B* genes in these cells (Figure 4C,D).

Consistent with these findings, bioinformatic analyses (https://www.genomaticx.de, accessed on 1 January 2021) identified a putative *HOXA5* binding site in both the *NFATC1* and *WNT2B* promoter regions (Figure S7), suggesting a direct transcriptional regulation of these genes by *HOXA5*.

As previously described [3], due to an inappropriate expansion and hypertrophy of their adipocytes, FDR individuals exhibit a SAT phenotype and metabolic profile similar to those of obese subjects, even if nonobese. Thus, to further investigate the relationship between a *HOXA5* deficit and dysfunctional SAT, we expanded our analysis and explored published large-scale human obesity expression profile datasets. Downregulation of *HOXA5* expression in whole abdominal SAT and in isolated SAT adipocytes from obese compared with lean individuals was observed (Figure S8). In this case, a negative correlation between the expression of the *WNT* target genes *NFATC1* and *WNT2B* and the expression of *HOXA5* also emerged (Figure S9), further supporting the suppressive action of *HOXA5* on these genes. Taken together, our findings and the in silico data strongly indicated that an *HOXA5* deficit is a distinctive feature of hypertrophic SAT, and that this deficit impairs SAT adipogenesis by inducing derangement in the *WNT*-signaling pathway.

### 3.4. HOXA5 Methylation Signature in Blood Leukocytes

Methylation changes detected in bloodborne DNA may represent a mark for other tissues more directly implicated in disease pathogenesis [25]. Accordingly, we also investigated whether the *HOXA5* promoter hypermethylation observed in the preadipocytes from FDR subjects also was reflected in similar changes in peripheral blood leukocytes (PBL).

**Figure 5:**
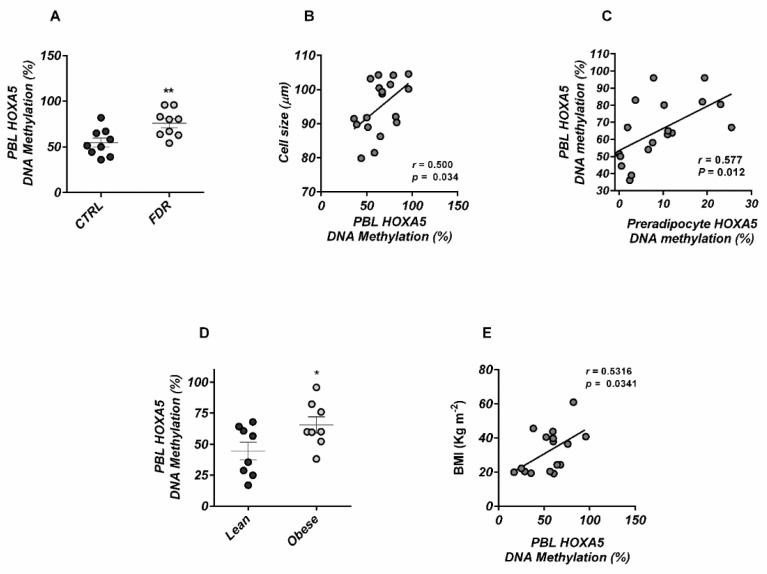
*HOXA5* DNA methylation in peripheral blood leukocytes from FDR and CTRL subjects. (**A**) Changes in average DNA methylation levels at 36 CpG within the *HOXA5* promoter were detected by BS analysis in PBL from FDR (n = 9) and CTRL (n = 9) subjects available from the study group. Mean values ± SD are also shown. ** *p* < 0.01 in a two-tailed Mann–Whitney U-test. (**B**) Correlation between the PBL *HOXA5* methylation and the mature adipose cell size or (**C**) with the preadipocyte *HOXA5* methylation in FDR (n = 9) and CTRL (n = 9) subjects available from the study group. The *r* correlation coefficients and *p*-values are indicated in the graph. HOXA5 methylation in peripheral blood leukocytes from obese and lean subjects. (**D**) BS analysis of the *HOXA5* promoter in PBL from obese (n = 8) and lean (n = 8) subjects from the replication cohort. Data points represent the DNA methylation percentage at the *HOXA5* promoter. Mean values ± SD are also shown. * *p* < 0.05 in a two-tailed Mann–Whitney U-test. (**E**) Correlation between the PBL *HOXA5* methylation and BMI in obese (n = 8) and lean (n = 8) subjects. The *r* correlation coefficient and *p*-value are indicated in the graph.

Bisulfite sequencing revealed that, similar to preadipocytes, PBL methylation levels at the *HOXA5* promoter were also increased in FDR individuals, as compared to subjects who were not FDR (Figure 5A). Importantly, the PBL hypermethylation at the *HOXA5* promoter positively correlated with subcutaneous adipose cell size in this study group (Figure 5B), and closely reflected the *HOXA5* methylation levels in the preadipocytes from each individual subject (Figure 5C). These findings suggested that PBLs represent a convenient and readily accessible proxy cell type for predicting the preadipocyte epigenetic signature at the *HOXA5* locus.

To further examine the impact of *HOXA5* dysregulation in metabolic health, we analyzed promoter methylation in a group of obese individuals (Table S3; a detailed description of this cohort is provided in [9,11]), since, very much like those who are FDR, obese subjects also feature inappropriately enlarged adipocytes in their SAT and a high risk of developing T2D [26,27]. Intriguingly, as in the case of the FDR, increased *HOXA5* promoter methylation was found in PBLs from the obese subjects (Figure 5D). *HOXA5* methylation levels in these subjects positively correlated with BMI (Figure 5E).

## 4. Discussion

Impaired adipogenesis in subcutaneous fat depots has been identified as a major pathogenetic factor in the progression toward type 2 diabetes [21]. In the present report, we have described a previously unrecognized role of the *HOXA5* transcription factor in regulating human adipogenesis. Previous studies have shown that in humans, *HOXA5* is highly expressed in different adipose tissue depots, and plays an active role in controlling both adipocyte function and body fat distribution [6,28]. In addition, we previously reported that *Hoxa5* silencing impaired adipocyte differentiation in mouse 3T3-L1 preadipocytes [7]. However, whether and how the *HOXA5* gene also plays a role in human adipogenesis have not been determined yet. The present work revealed that silencing of *HOXA5* in human preadipocytes impaired in vitro adipogenesis, as detected by reduced expression of adipocyte differentiation markers and decreased lipid droplet accumulation. Collectively, these data led us to conclude that not only *HOXA5* has a functional importance in human adipogenesis, but its requirement for adipose tissue differentiation is conserved from mouse to human.

The pathophysiological relevance of these in vitro results was further supported by our findings in individuals who were FDR of T2D patients. These individuals feature restricted adipogenesis [3], which rendered them of interest for investigating the role of *HOXA5* in human disorders. Subjects who are FDR of type 2 diabetics are at a high risk of developing this disorder, and feature dysfunctional subcutaneous adipose tissue accompanied by increased adipocyte cell size (hypertrophy) due to an impaired ability to recruit and/or differentiate new preadipocytes [3]. In preadipocytes from FDR individuals, we observed a significant reduction in the mRNA expression of *HOXA5* compared to individuals who were not FDR. In addition, we showed that the reduced expression of *HOXA5* in FDR negatively correlated with the SAT adipose cell hypertrophy caused by the impaired adipogenesis occurring in these individuals [29]. Thus, in preadipocytes from FDR individuals, the low *HOXA5* expression marks the impaired adipogenesis that renders their SAT dysfunctional.

*HOXA5* expression is epigenetically regulated in mammals [17]. Loss of *HOXA5* expression has been associated with increased methylation of its promoter in human breast and lung cancers [30,31]. In addition, we recently demonstrated that DNA hypermethylation at the *Hoxa5* promoter was accompanied by reduced expression in the adipose tissue of mice exposed to a high-fat diet [7]. Accordingly, in the present work, we tested the hypothesis that *HOXA5* downregulation in FDR individuals is determined by methylation abnormalities at this gene. Our data revealed that, in FDR preadipocytes, *HOXA5* repression was determined by increased methylation at its promoter. Indeed, luciferase assays demonstrated that methylation at this locus directly repressed the transcriptional activity of *HOXA5* in vitro, making it likely that the same occurs in vivo as well. All together, these findings supported the interpretation that a disturbed *HOXA5* methylation releases the brake on downstream target genes, enabling repression of adipocyte differentiation and contributing to SAT dysfunction in FDR. In line with these novel findings, we recently demonstrated that dysfunctional SAT and reduced adipogenesis in FDR subjects were associated with a differential methylation profile of genes relevant to adipocyte biology or adipogenesis in preadipocytes [9,21].

Coming to the mechanisms by which *HOXA5* regulates human adipogenesis, *Ordonez-Moran* et al. demonstrated an antagonistic relationship between *HOXA5* and the *WNT* signaling in colorectal cancer cells. In this paper, the authors reported that *HOXA5* inhibited *WNT*-induced transformation of intestinal cells, counteracted stemness programs, and enforced differentiation in normal tissue and intestinal cancer cells [22]. More recently, it was further reported that the ectopic expression of *HOXA5* suppressed proliferation and neoplasia in cervical cancer cells by repressing *Wnt* signaling [23]. The *WNT*-signaling pathway also was reported to play a pivotal role in the commitment and differentiation of adipose cells. Indeed, the activation of *WNT* signaling is known to prevent adipocyte differentiation [23]. Similar mechanisms occur in *HOXA5*-silenced preadipocytes, in which an *HOXA5* deficit appears to trigger dysregulation of the transcriptional program at several genes in the *WNT*-signaling pathway, ultimately leading to impaired adipogenesis. Indeed, our work revealed that transcription of a number of *WNT*-signaling genes was altered in the *HOXA5*-silenced preadipocytes. Some of these, including *FZD8*, *CSNK2A1*, *NFATC1*, *RHOA,* and *WNT2B*, are reportedly stimulated by and/or are key mediators of *WNT* signaling, and exhibited increased expression in preadipocytes in which *HOXA5* had been silenced. Taken together, these data indicated that, in human preadipocytes, *HOXA5* promoted adipogenesis by restraining *WNT* signaling.

Previous studies have shown that hypertrophy of mature adipocytes associates with an improper activation of the *WNT*-signaling pathway [32]. In fact, Henninger J. et al. reported increased *WNT*-signaling activity in adipose tissue from FDR compared to control subjects [24]. These observations, along with our findings in preadipocytes in which *HOXA5* had been silenced, prompted us to explore in detail the functional significance of the *WNT*-signaling cascade to the adipocyte hypertrophy observed in FDR individuals. We found that the expression of *NFATC1* and *WNT2B*, two key mediators of the *WNT* signal, was most robustly increased in the preadipocytes of FDR compared to control subjects. The expression of these two genes negatively correlated with that of *HOXA5* in the FDR subjects, suggesting a suppressive action of *HOXA5* on the transcriptional activity of *NFATC1* and *WNT2B*. Consistent with this possibility, subsequent in silico analysis revealed putative *HOXA5* binding sites in the promoter region of both the *NFATC1* and *WNT2B* genes.

Other investigators have previously shown that *NFATC1* and *WNT2B* feature antiadipogenic properties and perform specific roles in osteogenic differentiation. *NFATC1* regulates osteoblast bone formation in cooperation with the *Osterix* gene [33]. Similarly, *WNT2B* controls the osteogenic differentiation of bone marrow stromal cells by enhancing the expression of the key osteogenetic genes, *RUNX2* and *Osterix* [34]. Intriguingly, the *RUNX2* and *Osterix* genes were reported to restrain adipocyte differentiation [35,36]. Thus, the *HOXA5* downregulation occurring in FDR subjects leads to increased activity through the *WNT* pathway with enhanced osteogenic and restrained adipogenic signals, which contribute to restricting SAT adipogenesis.

Different authors have previously reported that DNA methylation in whole blood mirrors epigenetic changes occurring in tissues that are less accessible but have an important impact on disease pathogenesis [25,37,38]. We therefore tested whether the *HOXA5* methylation changes identified in SAT preadipocytes also reflected those occurring in bloodborne DNA. As hypothesized, *HOXA5* promoter methylation levels were very similar in FDR preadipocytes and PBLs obtained from the same individuals. Importantly, blood DNA methylation at the *HOXA5* locus positively correlated with the subcutaneous adipose cell size in FDR, in which adipocyte hypertrophy is both a marker of restricted adipogenesis and an independent predictor of T2D [9]. These latter findings led us to conclude that *HOXA5* DNA methylation in blood may represent a bona fide surrogate mark of preadipocyte epigenetic profile in FDR. Our study further demonstrated the significance of *HOXA5* methylation profile in blood as a mark of adipose tissue dysfunction and T2D risk, since methylation changes at this locus were replicated in an independent cohort of obese patients. Similar to FDR, these individuals, as a group, also featured dysfunctional subcutaneous fat and increased risk of developing T2D [1,23]. Blood methylation at the *HOXA5* promoter positively correlated with BMI in these obese individuals, indicating that *HOXA5* epigenetic profile also was associated with the level of adiposity in these subjects. In a previous study, association between bloodborne DNA methylation at *HOXA5* and BMI in a Mexican-American obese cohort was described [8]. Thus, in the future, validation of these findings may enable the identification of individuals at increased risk of unfavorable metabolic outcome, either due to diabetes familiarity or because of obesity.

In conclusion, this study revealed a previously unidentified function of *HOXA5* in maintaining adipogenesis in humans by restraining repressive signals from the *WNT* pathway. We further showed that *HOXA5*-altered DNA methylation and downregulation restricted adipogenesis in FDR of T2D patients, which may contribute, in part, to their increased risk of metabolic complications.

Exploitation of our findings may, in the future, reveal epigenetic changes contributing to obesity and T2D risk. Blood-based DNA methylation changes may further represent markers applicable to monitor, delay, or prevent the onset of T2D and obesity, and to facilitate early intervention strategies, particularly lifestyle-based treatment.

## Supplementary Materials

The following are available online at www.mdpi.com/article/10.3390/cells11040728/s1, Table S1: List of primers used in this study. Figure S1: HOXA5 silencing during in vitro adipogenesis of human SAT preadipocytes. Figure S2: The effect of HOXA5 silencing on the expression of preadipocyte and apoptosis markers. Table S2: Clinical characteristics of FDR and CTRL subjects. Figure S3: DNA methylation at the HOXA5 promoter in preadipocytes from FDR and CTRL subjects. Figure S4: The correlation between HOXA5 DNA methylation and its mRNA levels. Figure S5: Validation of the WNT-signaling pathway array results between the HOXA5-silenced and scrambled cells. Figure S6: The effect of HOXA5 silencing on the protein levels of WNT-signaling factors. Figure S7: In silico identification of putative HOXA5 binding sites across the promoter regions of NFATC1 and WNT2B genes. Figure S8: HOXA5 levels in whole abdominal SAT and in isolated SAT adipocytes from obese individuals. Figure S9: Correlation between NFATC1 and WNT2B expression and HOXA5 expression in isolated SAT adipocytes from obese individuals. Table S3: Clinical characteristics of obese and lean subjects.
