# Supplementary material for: Epigenetic Dysregulation of the Homeobox A5 (HOXA5) Gene Associates with Subcutaneous Adipocyte Hypertrophy in Human Obesity
Source: Cells. 2022 Feb 18;11(4):728. doi: 10.3390/cells11040728 (PMC8870634; doi:10.3390/cells11040728)
Supplement: Supplementary file 1 [file cells-11-00728-s001.zip › cells-1598432-supplementary.pdf]

Supplementary Table S1. List of primers used in this study.

GENE	*Forward/Reverse*	*Primer (5′ to 3′)*
HOXA5 Bisulphite	*Forward*	*TTTTGTATAATTTATGATGAATTATGGAAA*
	Reverse	*ACAAAAATAAAAAAAACTACAAAAC*
HOXA5 mRNA	*Forward*	*GCGCAAGCTGCACATAAG*
	Reverse	*CGGTTGAAGTGGAACTCCTT*
GLUT4 mRNA	*Forward*	*TCTGGCATCAATGCTGTTTTCTAT*
	Reverse	*ACCAACAACACCGAGACCAAG*
FABP4 mRNA	*Forward*	*GACAGGAAAGTCAAGAGCACCATA*
	Reverse	*GACGCATTCCACCACCAGTT*
RPL13A mRNA	*Forward*	*CTTTCCGCTCGGCTGTTTTC*
	Reverse	*GCCTTACGTCTGCGGATCTT*
ADIPONECTIN mRNA	*Forward*	*CCGGGCCATAATGGGG*
	Reverse	*CCCTTAGGACCAATAAGACCTGG*
WNT2B mRNA	*Forward*	*CGAGAGTGTCAGCACCAATTC*
	Reverse	*AAGCTGCCTCTCGGCTACT*
FZD8 mRNA	*Forward*	*CACCGTCTCCACCTTCCTTA*
	Reverse	*GACACGAAGAGGTAGCAGGC*
CSNK2A1 mRNA	*Forward*	*GAACGCTTTGTCCACAGTGA*
	Reverse	*TATCGCAGCAGTTTGTCCAG*
NFATC1 mRNA	*Forward*	*CACCGCATCACAGGGAAGAC*
	Reverse	*GCACAGTCAATGACGGCTC*
ROHA mRNA	*Forward*	*AGCCTGTGGAAAGACATGCTT*
	Reverse	*TCAAACACTGTGGGCACATAC*
FOSL1 mRNA	*Forward*	*CAGTGGATGGTACAGCCTCA*
	Reverse	*CAGTTTGTCAGTCTCCTGTTCAC*
GATA2 mRNA	*Forward*	*CAGCAAGGCTCGTTCCTGTTCA*
	Reverse	*ATGAGTGGTCGGTTCTGCCCAT*
GATA3 mRNA	*Forward*	*TCATTAAGCCCAAGCGAAGG*
	Reverse	*GTCCCCATTGGCATTCCTC*
FAS mRNA	*Forward*	*ACCCTTGCACCAAATGTGAAC*
	Reverse	*AAGAAGACAAAGCCACCCCA*
HOXA5 Cloning	*Forward*	*CGCCCTAGGGTCTATAGCACCCTTGCACA*
	Reverse	*CGCGGATCCGCTGCAAAACGGGGGAA*

Supplementary Figure S1

***HOXA5* silencing during *in vitro* adipogenesis of human SAT preadipocytes.** *HOXA5* protein levels were measured by western blot analysis at different time points during differentiation in cells treated with *HOXA5*-specific siRNA, Scramble siRNA (*SCR*) or the control (*CTRL*). Vinculin served as a loading control. The upper figure shows representative blots; the lower figure presents the results quantification, where the black dotted line represents control un-transfected cells. The results are the mean ± SD of three independent experiments. The statistical significance was tested by one-way ANOVA followed by Tukey's multi-comparison test at each indicated time point: *p<0.05, **p<0.01, ***p<0.001 for comparison *versus CTRL*; ##p<0.01, ###p<0.001 *versus SCR*.

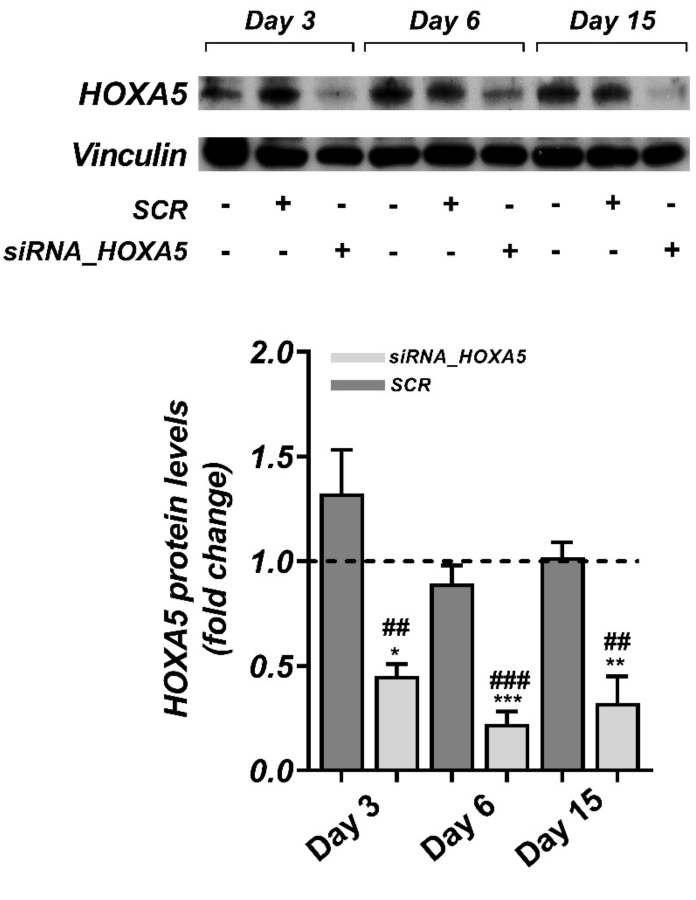

Supplementary Figure S2

The effect of *HOXA5* silencing on the expression of preadipocyte and apoptosis markers. The expression levels of *GATA2*, *GATA3* and *FAS* were assessed by qPCR at different time points during differentiation in the cells treated with scramble (SCR) or *HOXA5*-specific siRNA (*siRNA_HOXA5*). All data are presented as means ± SD of 3 independent experiments. Statistical significance was tested by one-way ANOVA followed by Tukey's multi-comparison test at each indicated time. **p*<0.05 *versus* SCR. N.S., no significant difference.

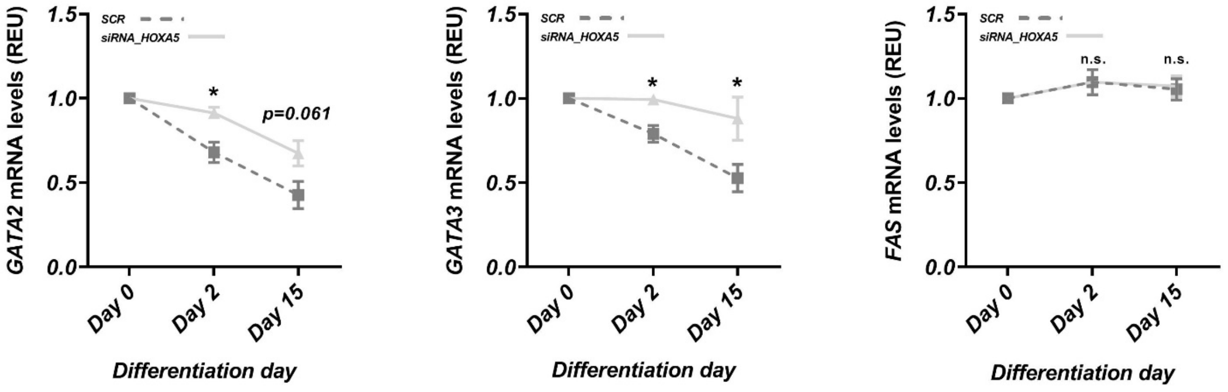

Supplementary Table S2. Clinical characteristics of FDR and CTRL subjects.

Measure	*FDR*	*CTRL*
N (female/male)	*12 (6/6)*	*12 (6/6)*
Age, years	*42.3 ± 2.9*	*38.7 ± 2.3*
BMI, Kg/m²	*25.4 ± 0.5*	*24.5 ± 0.7*
Fat percent, %	*26.9 ± 2.4*	*24.0 ± 1.9*
Waist to Hip Ratio (WHR)	*0.90 ± 0.02***	*0.81 ± 0.02*
Total triglycerides, mmol/L	*1.5 ± 0.6***	*0.8 ± 0.2*
HDL cholesterol, mmol/L	*1.3 ± 0.2**	*1.6 ± 0.3*
Cell size, µm	*100.2 ± 1.7****	*89.4 ± 1.9*
GIR/bw, mg/Kg/min	*7.9 ± 0.6***	*11.5 ± 0.8*
f-insulin, pmol/L	*60.1 ± 8.0***	*35.4 ± 3.9*
fb-glucose, mmol/L	*4.8 ± 0.1***	*4.4 ± 0.1*
OGTT p-glucose 2h, mmol/L	*6.6 ± 0.6**	*4.8 ± 0.4*
HOMA-IR index	*2.0 ± 0.8****	*1.1 ± 0.4*

Clinical characteristics of FDR and CTRL subjects. The study group has been described under Materials and Methods. All data are expressed as means ± SD. Statistical significance was tested by a two-tailed Mann-Whitney U-test: **p<0.05, **p<0.01, ***p<0.001,* FDR *vs.* CTRL. BMI, body mass index; GIR/bw, glucose infusion rate/body weight; OGTT, oral glucose tolerance test; HDL, high density lipoprotein; HOMA-IR, Homeostatic Model Assessment of Insulin Resistance.

Supplementary Figure S3

A

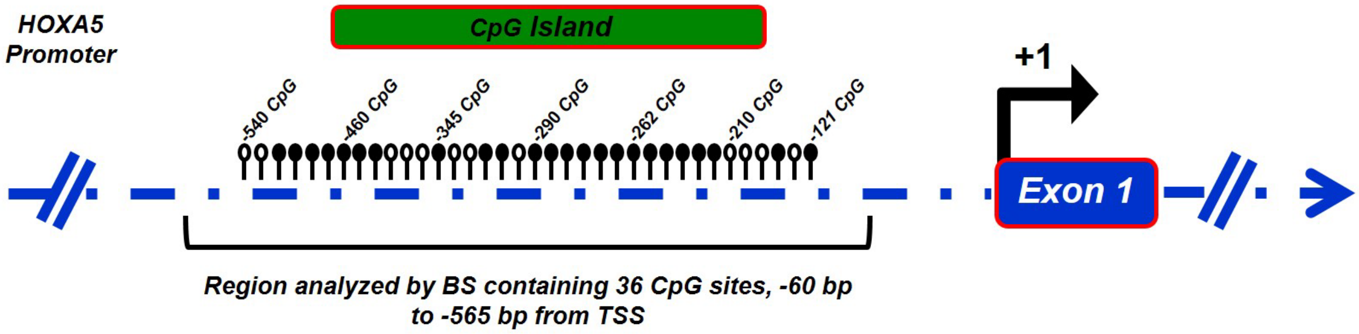

B

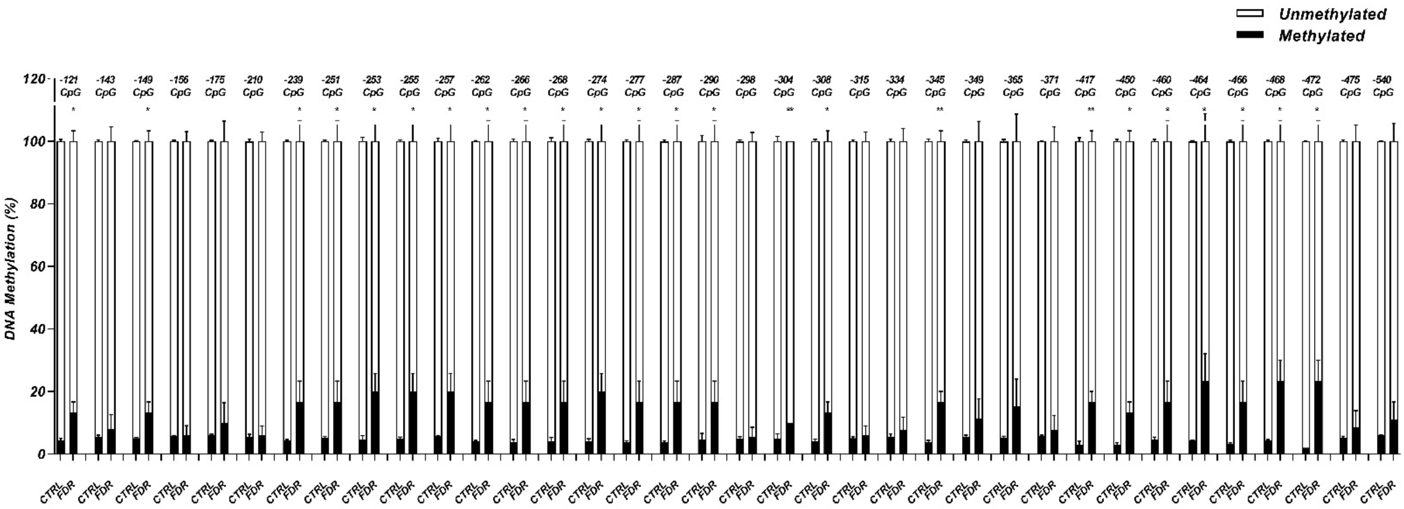

Supplementary Figure S4

The correlation between *HOXA5* DNA methylation and its mRNA levels. The correlation between *HOXA5* DNA methylation and *its* mRNA levels in preadipocytes from FDR (n=9) and CTRL (n=9) subjects available from the study group. The r correlation coefficient and p-value are indicated in the graph.

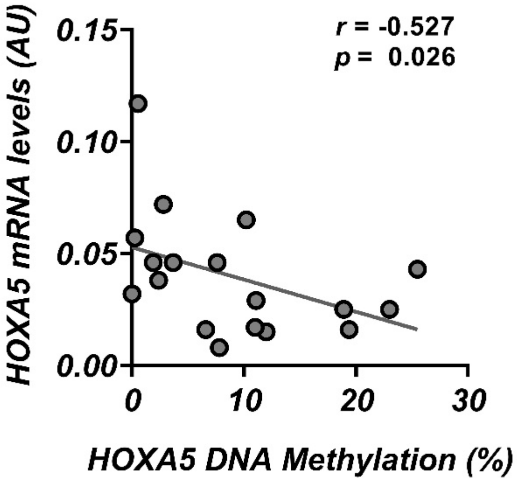

Supplementary Figure S5

Validation of the *WNT*-Signalling Pathway array results between the *HOXA5*-silenced and scrambled cells. The 11 differentially expressed genes, identified by the RT2 Profiler™ PCR array, were individually analysed by qPCR in the same samples also tested in the PCR array (n = 3 per group/condition; please also refer to Figure 3 of the main text). Five of the 11 differentially expressed genes were not confirmed by qPCR assay. All the data are expressed as means ± SD. N.S., no significant difference.

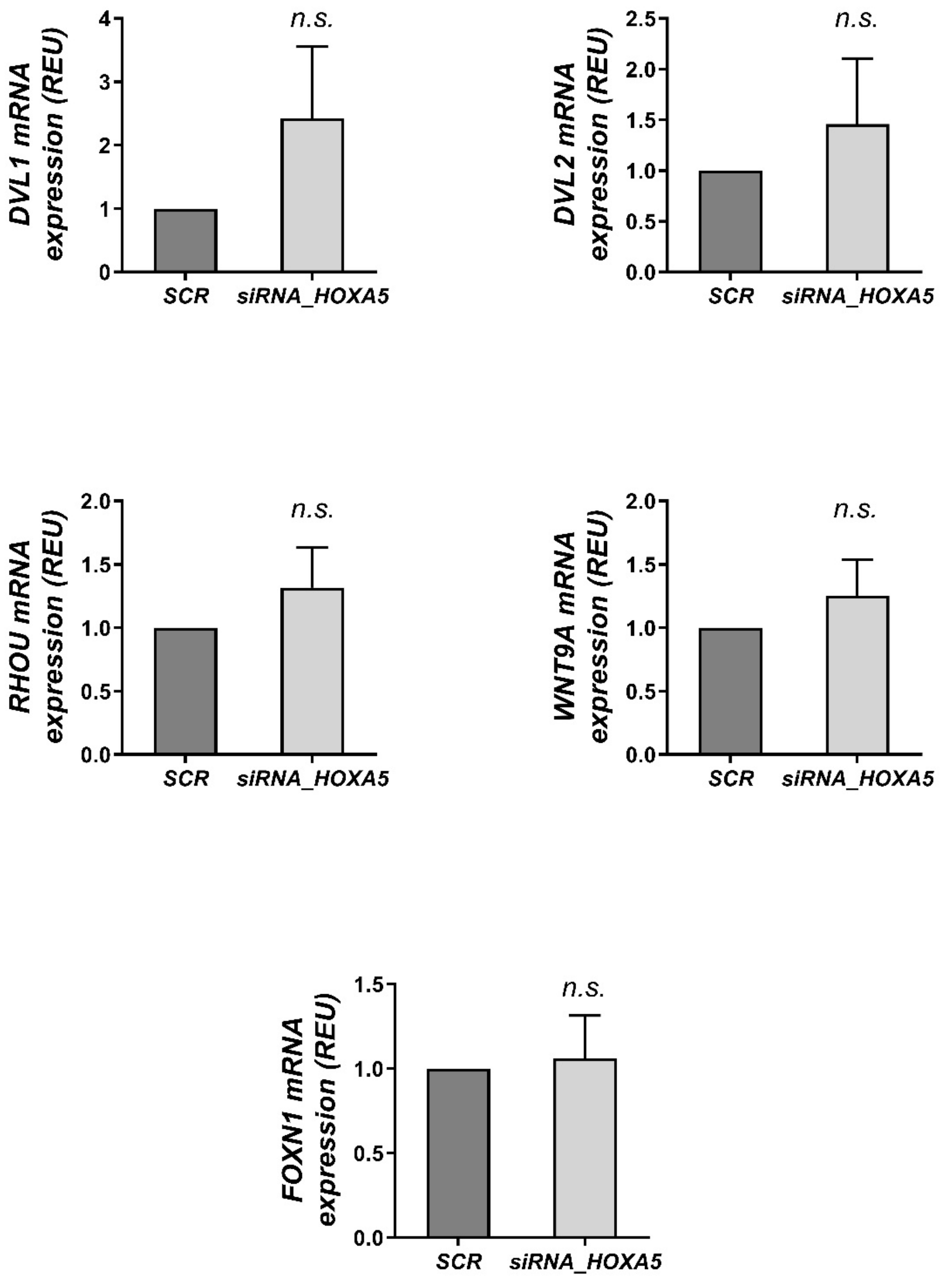

Supplementary Figure S6

The effect of *HOXA5* silencing on the protein levels of *WNT*-Signalling factors. The *HOXA5*, *WNT2B* and *NFATC1* protein levels were measured by western blot analysis in preadipocytes treated with *HOXA5*-specific siRNA (*siRNA_HOXA5*), Scramble siRNA (SCR) and in control un-transfected cells. Vinculin served as a loading control. The upper figure shows representative blots; the lower figure presents the results quantification, where the black dotted line represents control un-transfected cells. The results are the mean ± SD of three independent experiments. The statistical significance was tested by one-way ANOVA followed by Tukey's multi-comparison test: *p<0.05 and **p<0.01 versus control un-transfected cells; #p<0.05 and ##p<0.01 versus *SCR*.

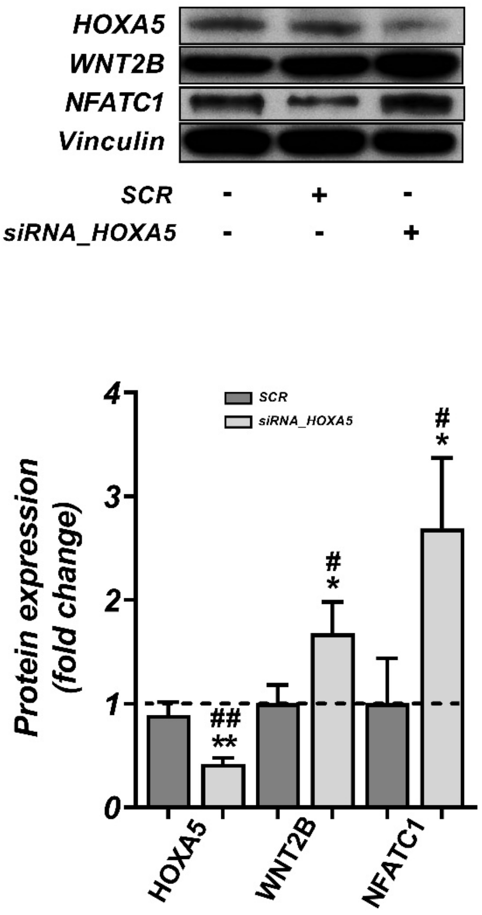

Supplementary Figure S7

***In silico* identification of putative *HOXA5* binding sites across the promoter regions of *NFATC1* and *WNT2B* genes.** The promoter region of both the *NFATC1* and *WNT2B* genes (1500 bp upstream the transcription start site; TSS) was analyzed in the *Genomatix Database* (https://www.genomaticx.de/) using the *MatInspector* platform. One putative *HOXA5* binding motif was identified in the *NFATC1* promoter at -957 bp upstream the TSS one further motif in the *WNT2B* promoter at -171 bp upstream the TSS.

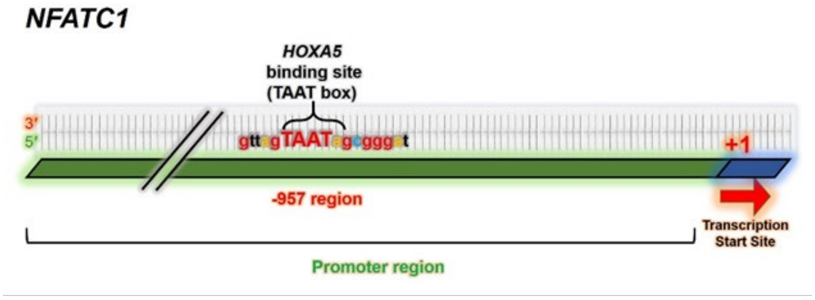

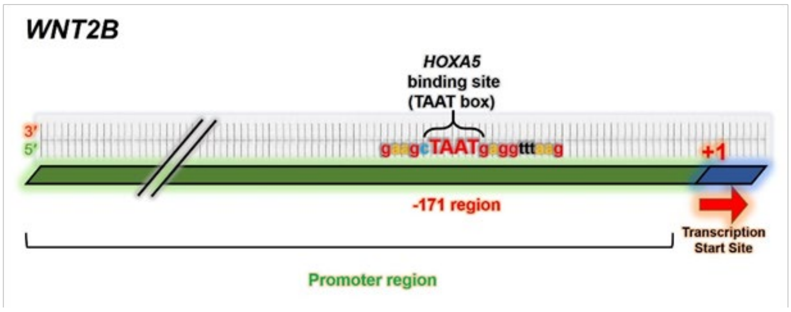

Supplementary Figure S8

***HOXA5* levels in whole abdominal SAT and in isolated SAT adipocytes from obese individuals.** Normalized mRNA expression of *HOXA5* in human abdominal SAT (**a**) and isolated SAT adipocytes (**b**) from obese and lean individuals. The data were obtained from large-scale human obesity expression datasets published in Gene Expression Omnibus (GEO). GEO accession number: GSE27951, (**a**), and GEO: GSE2508, (b). ***p<0.05, **p<0.01* **in a two-tailed Mann–Whitney U-test.**

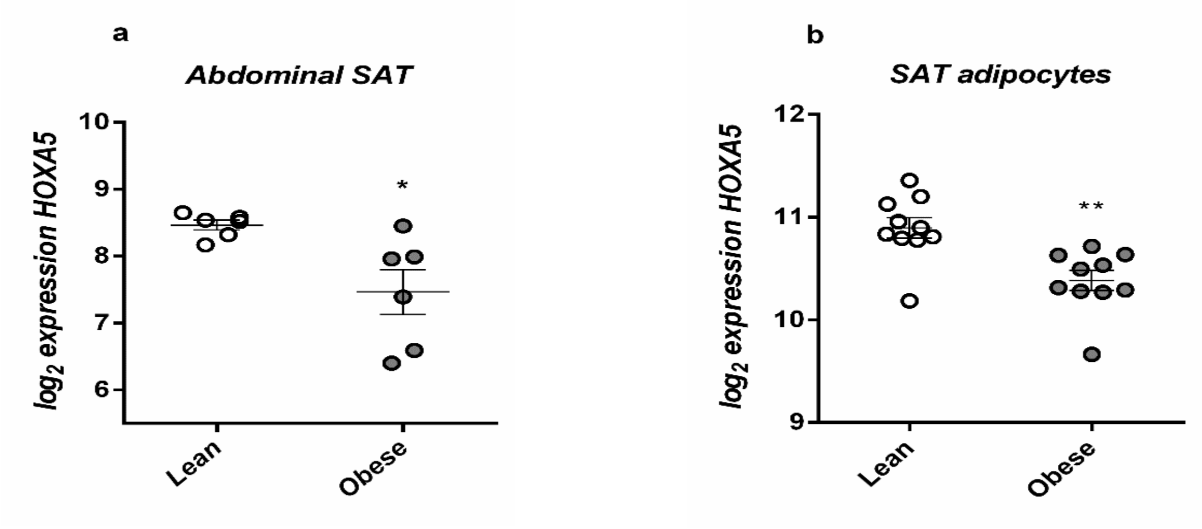

Supplementary Figure S9

Correlation between *NFATC1* and *WNT2B* expression and *HOXA5* expression in isolated SAT adipocytes from obese individuals. The data were obtained from large-scale human obesity expression datasets published in Gene Expression Omnibus (GEO). GEO accession number: GSE2508. **The *r* correlation coefficients and *p-values* are indicated in the graph.**

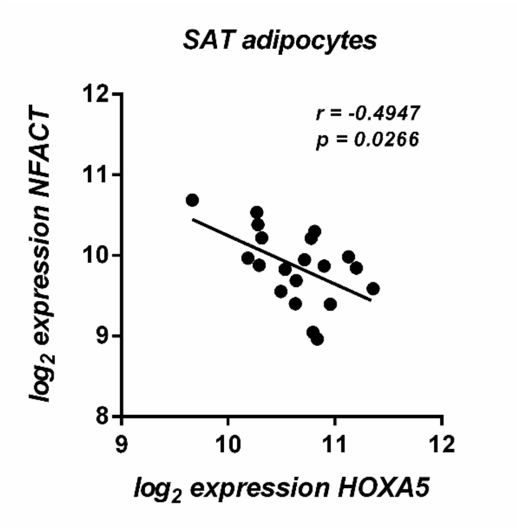

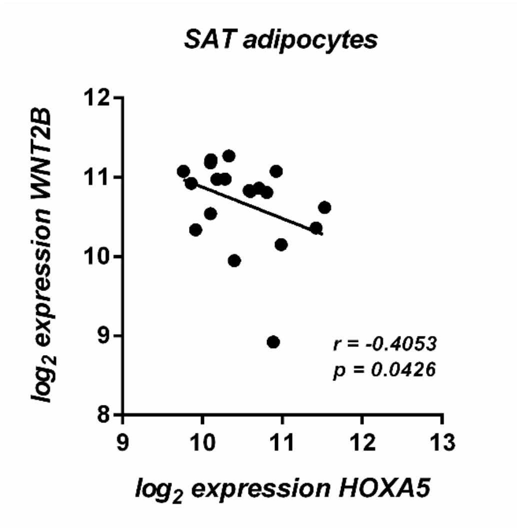

Supplementary Table S3. Clinical characteristics of Obese and Lean subjects.

Measure	*Obese*	*Lean*
N (female/male)	*8 (4/4)*	*8 (4/4)*
Age, years	*36.2 ± 5.3**	*31.3 ± 2.1*
BMI, Kg/m^2	*43.0 ± 6.9****	*22.2 ± 2.9*
Total triglycerides, mmol/L	*1.6 ± 0.6****	*0.7 ± 0.2*
HDL cholesterol, mmol/L	*1.3 ± 0.3**	*1.8 ± 0.5*
fb-glucose, mmol/L	*4.7 ± 0.3*	*4.9 ± 0.4*

Clinical characteristics of Obese and Lean subjects. All data are expressed as means ± SD. Statistical significance was tested by a two-tailed Mann-Whitney U-test: *$p<0.05$, ***$p<0.001$, Obese *vs.* Lean. BMI, body mass index; HDL, high density lipoprotein.
